# 

**Published:** 1915-03-06

**Authors:** 


### CARDIFF'S OLDEST ACTIVE MEDICAL PRACTITIONER.

Dr. John Evans, of Canton, Cardiff, who died on February 15, was Cardiff's oldest active medical practitioner upon his retirement in March last. He was born in London, and since 1870 had lived continuously in Cardiff. He proceeded to St. Bartholomew's Hospital in 1863, and took the diplomas of M.R.C.S. and L.S.A. Dr. Evans had been medical officer for the Llandaff and Canton district, and public vaccinator. He had also been medical officer to the Ely Schools and various children's homes, and was formerly a member of the City Council.

### MEDICAL SALARIES AND THE WAR.

The Camberwell Board of Guardians have decided to increase the remuneration for a temporary resident medical officer at Constance Road Institution to £300 a year, with the usual indoor allowances, valued at £90 per annum. The board has had considerable difficulty in filling the post, Dr. F. T. D. Clindening, who was recently appointed, having tendered his resignation on being called upon to join the Medical Corps at the Front.

### Medical Appointments.

Dr. D. W. Griffith, M.R.C.S. Eng., L.R.C.P. Lond., has been appointed by the Metropolitan Asylums Board as a second assistant medical officer in the Asylums Service, whilst Mr. T. J. Lydon and Mr. H. J. Phillips have been appointed assistant medical officers in the Infectious Hospitals Service.

Mr. N. K. Foster, fourth assistant medical officer at Horton Mental Hospital, has been appointed third assistant medical officer, and Mr. R. G. Riches, fifth assistant medical officer, has been promoted to be fourth assistant.

### Personalia.

Dr. E. E. Fremantle, the county medical officer of health and chief school medical officer for Hertfordshire, has been promoted from the rank of Major to the temporary rank of Lieutenant-Colonel in the R.A.M.C.(T.) as from the 14th ult. Lieutenant-Colonel Fremantle is serving as divisional sanitary officer to the East Anglian Division T.F. at Bury St. Edmunds.

Dr. F. T. D. Clindening, temporary resident medical officer at the Constance Road Institution, Camberwell, has resigned his temporary appointment as resident medical officer. His services have been required by the military authorities in connection with the Royal Army Medical Corps. Before advertising for a successor the Camberwell Board of Guardians have decided to take into consideration the remuneration attached to the office.

### Buildings Contemplated.

Barnsley.—The Corporation propose to carry out extensions at the Hendray Hospital.

Little Hulton.—About £7,000 will be spent on the adoption of Peel Hall as a sanatorium.

Milford.—The Surrey County Council have authorised the Public Health Committee to proceed with their preliminary inquiries and preparation of plans and estimates for a sanatorium and hospital on the Sattenham estate, Milford.

Mossley.—The Mossley Sick Nursing Association are considering the building of a new hospital.

Netley.—The Executive Committee of the Welsh War Hospital at Netley have decided to add 100 beds to the existing accommodation. The additional buildings, by Messrs. Edwin Hall and E. Stanley Hall, hon. architects of the hospital, will be started at once.

Nuneaton.—The House Committee recommend the Guardians to prepare a scheme for new receiving wards and isolation wards and for better accommodation for female tramps and children.

Scalebor.—At a meeting of the West Riding County Council at Wakefield, it was decided to purchase additional property on the Scalebor Park Asylum estate at a cost of £6,000, with a view to providing accommodation for wealthy patients.

Stafford.—The Staffs, Wolverhampton, and Dudley Joint Tuberculosis Committee have received the approval of the Local Government Board to the purchase of Groundslow House for conversion into a sanatorium. Tenders will be obtained for the work.

Stourbridge.—The new tuberculosis pavilion which has been built by the Stourbridge and Halesowen Hospital Committee at the Hayley Green Hospital has been formally opened. The cost was £2,214.

St. Pancras.—The Guardians have adopted a scheme for providing accommodation for about 200 additional male inmates at the workhouse, at an estimated cost of £550. Mr. A. E. Pridmore, architect, has been asked to report on the practicability of the scheme.

Rates of Subscription: Twelve months, 6/6; Six months, 4/-; Three months, 2/-, post free to any address in the United Kingdom. Abroad, Twelve months, 8/8; Six months, 5/-; Three months, 2/6, post free.—Address The Subscription Dept., "The Hospital," The Hospital Building, 28/29 Southampton Street, Strand, London, W.C.

